# A case report of a novel approach to the management of refractory coronary vasospasm

**DOI:** 10.1093/ehjcr/ytaf500

**Published:** 2025-10-03

**Authors:** Danielle Lapin, Con Arronis, Nigel Jepson

**Affiliations:** Cancer Centre for Children, The Children’s Hospital at Westmead, Hawkesbury Rd, Westmead, NSW 2145, Australia; Department of Cardiology, Prince of Wales Hospital, High St, Randwick, NSW 2031, Australia; Department of Cardiology, Prince of Wales Hospital, High St, Randwick, NSW 2031, Australia

**Keywords:** Case report, Refractory coronary vasospasm, Corticosteroids, Wearable cardiac defibrillator

## Abstract

**Background:**

Coronary vasospasm, or vasospastic angina (VSA), is a clinical entity that can uncommonly occur in the setting of non-obstructive coronary disease. Refractory VSA can be associated with significant adverse cardiovascular events, and a paucity of evidence-based guidelines makes this a challenging clinical scenario for clinicians to treat effectively.

**Case summary:**

A 46-year-old male presenting with a 48 h history of epigastric pain with electrocardiogram changes suggestive of left anterior descending artery stenosis and angiography showing no significant obstructive disease. The patient then developed recurrent early morning chest pain with multi-territory ST elevation despite antispasm therapy with repeat angiography and optical coherence tomography showing no evidence of obstructive disease. The patient was diagnosed with idiopathic refractory coronary vasospasm and ultimately managed with corticosteroids and with a wearable cardiac defibrillator.

**Discussion:**

The case presented highlights the diagnostics challenges and treatment complexity in managing refractory coronary vasospasm in which our patient experienced a good long-term outcome despite high risk features. Steroids are infrequently used in the management of coronary vasospasm but represent a life-saving treatment option in the setting presumed inflammation. Wearable cardiac defibrillator can be used to both investigate and prevent fatal arrhythmia and should be considered in high-risk coronary vasospasm cohorts.

Learning points

- Refractory coronary vasospasm is a broad clinical entity with a variety of underlying aetiologies that can present difficulties in elucidating the underlying diagnosis.
- Corticosteroids can represent a life-saving treatment in patients with suspected vasculitides or inflammation manifesting as idiopathic refractory coronary vasospasm.
- Wearable cardiac defibrillators may have a role in preventing cardiac vasospasm related morbidity.

## Introduction

In coronary vasospasm, or vasospastic angina (VSA), transient coronary artery constriction can produce life-threatening myocardial ischaemia without significant coronary artery atherosclerosis. The acute coronary syndrome (ACS) may go unrecognized due to patients lacking classical risk factors or common cardiac findings.^1,2^ Corticosteroids are recognized in treating refractory VSA where inflammation is suspected as the underlying mechanism.^2–4^ There is a paucity of evidence, including no large scale trials, supporting using corticosteroids in refractory vasospasm without proven inflammation. Our case highlights corticosteroids as a life-saving treatment modality in idiopathic refractory VSA in a patient experiencing recurrent early morning multi-territory vessel ischaemia.

## Summary figure

**Figure:**
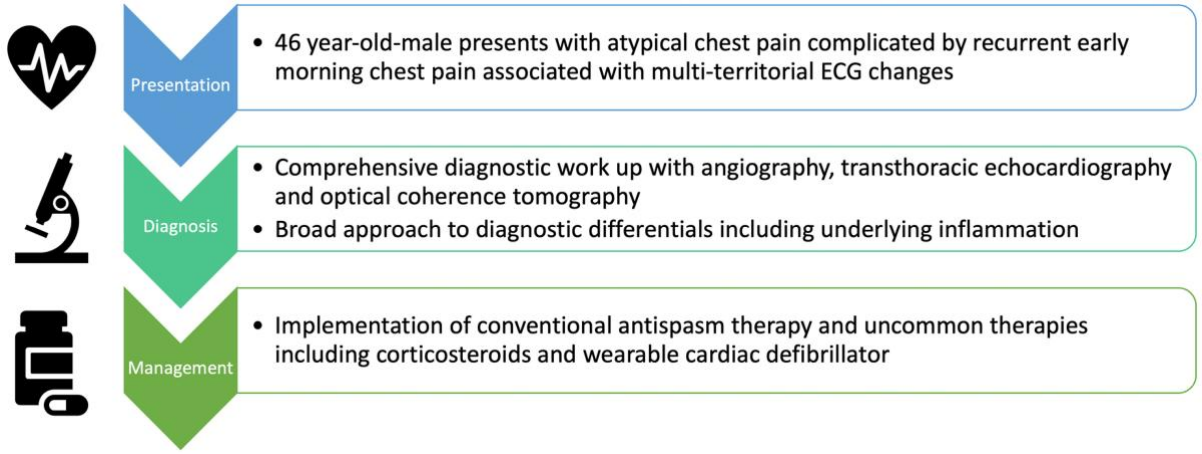


## Case presentation

**Figure 1:**
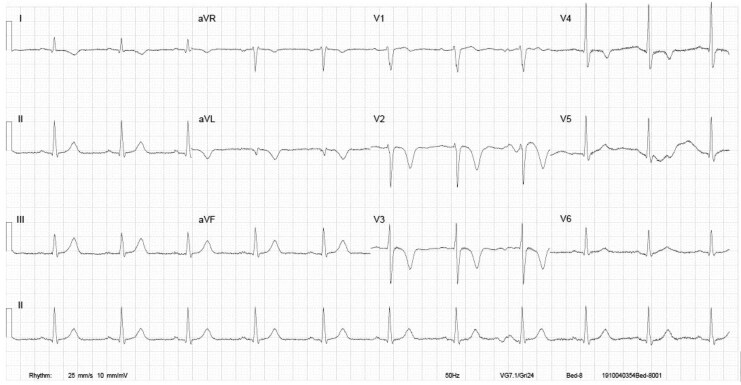
Initial electrocardiogram recorded in the emergency department. Electrocardiogram shows T wave inversion present in V2–V3 suggestive of critical left anterior descending stenosis.

**Figure 2:**
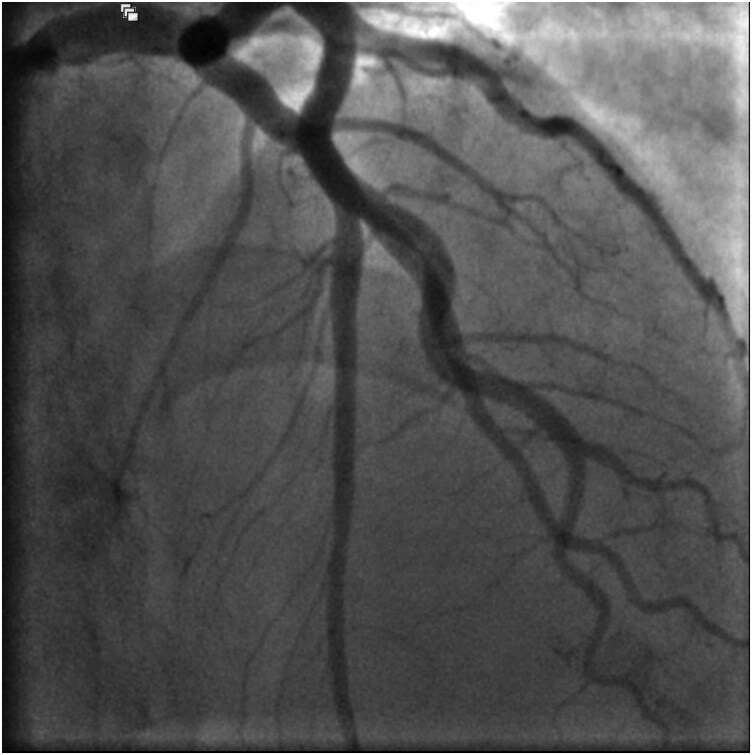
The image demonstrates the left anterior descending artery taken from coronary angiography during the index procedure and shows mild, non-obstructive coronary disease of the proximal to mid vessel.

A 46-year-old man of Chinese descent presented to the emergency department with 48 h of epigastric pain. He is an ex-smoker with a strong family history of coronary disease but no history of illicit substance use or remarkable clinical findings. Electrocardiogram (ECG) demonstrated T wave inversion in leads V2–3 suggestive of critical left anterior descending (LAD) stenosis (*Figure 1*). High-sensitivity troponin-T level was initially 343 ng/L (high > 14 ng/L) and 408 ng/L on repeat sequence. Transthoracic echocardiography (TTE) showed normal left ventricular (LV) size with mild to moderate segmental systolic impairment and apical hypokinesis consistent with LAD disease. Coronary angiography demonstrated mildly irregular, large calibre vessels but no severe stenoses (*Figure 2*). Dual antiplatelet (DAPT) therapy was initiated with loading doses pre-procedure and continued post-procedure. Rosuvastatin 20 mg daily was added due to the unclear aetiology of the ACS. Heparin was discontinued.

**Figure 3:**
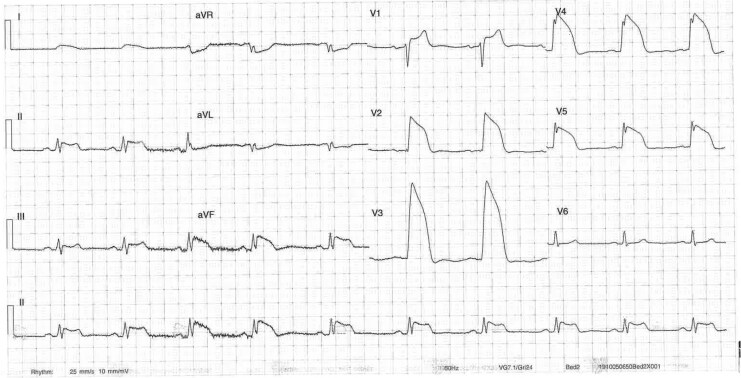
Electrocardiogram on Day 2 of admission during episode of central chest pain demonstrating significant anterior and inferior ST elevation.

**Figure 4:**
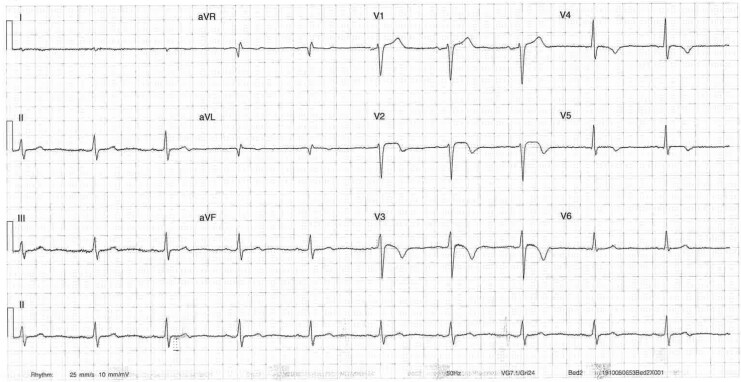
Electrocardiogram on Day 2 of admission following administration of glyceryl trinitrate 3 min after the electrocardiogram in *Figure 3* during episode of acute chest pain.

**Figure 5:**
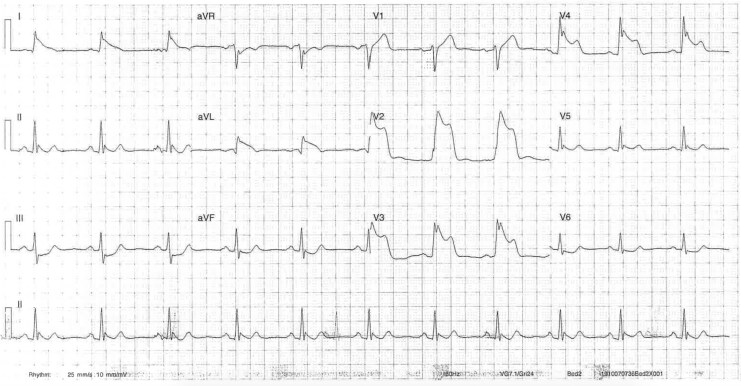
Electrocardiogram on Day 4 of admission during episode of central chest pain demonstrating anterior and lateral ST elevation.

Early morning on Day 2 of admission, the patient experienced unprovoked sudden onset central chest pain. Electrocardiogram showed extensive anterior and inferior ST elevation (*Figure 3*) consistent with multi-territory ischaemia and pain resolved following 300 mcg of sublingual glyceryl trinitrate (GTN) (*Figure 4*). Urgent repeat coronary angiography showed unchanged mild non-obstructive disease. Intra-coronary imaging of the LAD artery with optical coherence tomography (OCT) performed revealed diffuse intima-medial thickening with homogenous fibrotic plaque throughout the middle and proximal vessel. There was minimal lipidic plaque with no intra-luminal thrombus, evidence of plaque rupture, or erosion, and presumptive diagnosis of coronary vasospasm was established. The patient commenced isosorbide mononitrate (ISMN) modified release (MR) 60 mg daily, nifedipine MR 30 mg daily, and magnesium 500 mg BD (twice daily). On Day 4, the patient had repeat unprovoked early morning central chest pain relieved by GTN administration. Electrocardiogram again demonstrated multi-territory ischaemia with anterior and lateral ST elevation (*Figure 5*). Isosorbide mononitrate MR increased to 120 mg daily and nifedipine MR increased to 30 mg BD.

Comprehensive vasculitic screen including CRP, ESR, rheumatoid factor, cyclic citrullinated peptide, ANA, ENA, ANCA, ds DNA, cardiolipin antibodies, beta 2 glycoprotein antibodies, and HIV/hepatitis screen was normal. Absolute eosinophils were normal at 0.16 × 10^9^/L (normal range 0.04–0.44 × 10^9^/L). Urine drug screen was normal. Computed tomography coronary angiogram (CTCA) investigating for atherosclerotic disease showed minimal circumferential narrowing of the right coronary artery but no lipid rich or fibro-fatty plaque suggestive of atherosclerosis. Positron emission tomography (PET)/CT scan showed no medium to large vessel vasculitis.

The utilization of corticosteroids was considered in our patient due to the life-threatening symptoms of the idiopathic refractory coronary vasospasm persisting despite escalating antispasmodic therapy. The suggestion of endothelial dysfunction on OCT imaging and lack of evidence of atherosclerosis suggested inflammation as a possible driver of the VSA, further supporting the rationale for corticosteroids. Following a 13-day admission, the patient was discharged on oral prednisone 25 mg daily, weaned to 17.5 mg and then 7.5 mg at 2 week intervals before ceasing. Previously established medications were continued on discharge. The patient was prescribed a wearable cardiac defibrillator (Lifevest, Zoll) to mitigate against spasm mediated sudden cardiac death (SCD) in the context of the high-risk ECG changes associated with pain. Cardiac vest was removed at the 3-month follow-up after the patient remained free of chest pain with TTE normalization of LV systolic function. Dual antiplatelet was continued to 18 months with aspirin only and other spasm therapies continued long term following discussion with the patient. The patient remained free of cardiac symptoms and events on serial review at 48 months.

## Discussion

The Coronary Vasomotor Disorders International Study Group’s diagnostic criteria for VSA are nitrate-responsive angina, transient ischaemic ECG changes, and coronary artery vasospasm.^5^ Our patient was classified as refractory without requiring provocative coronary spasm testing due to events not relieved or prevented by at least two coronary vasodilatory agents.^6^ The incidence of SCD increases with features exhibited by our patient which were multi-vessel spasm, LAD coronary artery involvement, and refractory vasospasm.^1,6^ Optical coherence tomography provides high spatial resolution assessment of coronary arteries including plaque rupture, erosion, thrombus, and atherosclerosis occurring at spasm sites that appear normal angiographically.^7^ Luminal irregularity is observed in a majority of spasm sites with no apparent thrombus on OCT imaging,^7^ consistent with our case and suggestive of possible inflammation.

Corticosteroid administration in coronary vasospasm has not been investigated in sufficiently powered randomized controlled trials, but a meta-analysis exploring corticosteroid use in ACS has suggested possible mortality improvement. Benefit was attributed to corticosteroids reducing myocardial infarction by diminishing the effects of harmful inflammation.^8^ Several case reports have highlighted the success of immunosuppressive therapy in controlling spasm events refractory to common vasodilators emphasizing the role of inflammation in propagating coronary spasm.^2,9,10^ Vasospasm in vasculitis occurs due to endothelial and vascular smooth muscle dysfunction that disrupts the balance between vasodilation and vasoconstriction activity.^9^ Corticosteroids in combination with cyclophosphamide have a recognized role in the treatment of coronary vasculitis in polyarteritis nodosa.^11^ Vasospastic angina is also related to eosinophils releasing vasospastic mediators in patients with eosinophilia with ischaemic chest pain with normal cardiac work up on angiogram and CTCA.^2^ In a series of four patients, localized inflammation and refractory VSA was demonstrated in all patients without the development of eosinophilia and steroid therapy was recommended in this cohort.^12^ Corticosteroid treatment was implemented in our case of idiopathic coronary vasospasm with no robust evidence guiding treatment but a postulated inflammatory component. Therapy controlled acute phase symptoms and prevented longer term recurrence. Our case suggests a broader role for corticosteroids in refractory VSA where autoimmune or vasculitic causes have not been identified.

There are no accepted guidelines for clinical implantation of automatic implantable cardiac defibrillators (AICD), but the role for primary prevention for SCD has been discussed in high-risk VSA population groups. Several reports have highlighted the role and benefits of a wearable cardiac defibrillator in patients with documented VSA perceived to be at high risk of ventricular arrhythmias and SCD.^6,13^ Wearable cardiac defibrillator has been shown to detect ventricular fibrillation associated with syncope in a case subsequently shown to be due to coronary vasospasm after which an AICD was inserted^14^ A prescription for a wearable cardiac defibrillator was given based on the patient’s high-risk arrhythmogenic state with recurrent ST elevation refractory to conventional therapy. The decision to opt for a vest and not proceed directly to AICD insertion allowed for a period of therapy with corticosteroids to resolve the underlying vasospastic process. Following 3 months of being symptom free, the patient was able to be re-stratified to a lower risk group and the vest removed.

## Lead author biography


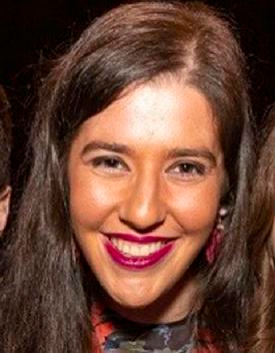


Dr Danielle Lapin is an advanced trainee through the Royal Australasian College of Physician (RACP) currently completing her sub-specialty in general paediatrics and paediatric oncology.

## Data Availability

No new data was generated or analysed in support of this research.
